# Digital Transition and Mental Health in School Settings: Does Handwriting Still Matter? A Multidisciplinary Perspective

**DOI:** 10.3390/children13070940

**Published:** 2026-07-17

**Authors:** Giuseppe Marano, Oksana Di Giacomi, Senad Hasaj, Gianandrea Traversi, Osvaldo Mazza, Andrea Cangini, Marianna Mazza

**Affiliations:** 1 Department of Neuroscience, Head-Neck and Chest, Section of Psychiatry, Fondazione Policlinico Universitario Agostino Gemelli IRCCS, Largo Agostino Gemelli 8, 00168 Rome, Italy; oksanadigiacomi@gmail.com (O.D.G.); senadhasaj98@icloud.com (S.H.); 2 Department of Neuroscience, Section of Psychiatry, Università Cattolica del Sacro Cuore, 00168 Rome, Italy; 3 Accademia di Psicologia e Espressione Della Scrittura, 00168 Rome, Italy; 4 Unit of Medical Genetics, Department of Laboratory Medicine, Ospedale Isola Tiberina-Gemelli Isola, 00186 Rome, Italy; gianandrea.traversi@gmail.com; 5 Spine Surgery Department, Bambino Gesù Children’s Hospital IRCCS, 00168 Rome, Italy; osvaldo.mazza1973@hotmail.it; 6 Fondazione Luigi Einaudi, 00193 Rome, Italy; 7 Osservatorio Carta, Penna & Digitale, 00193 Rome, Italy

**Keywords:** handwriting, children, mental health, neurodevelopment, learning processes, emotional regulation, digital education, developmental psychology, child psychiatry, school environment

## Abstract

**Highlights:**

**What are the main findings?**

- Handwriting recruits fine motor, visuomotor, cognitive, and language-related systems that support literacy, memory, conceptual learning, and self-reflection.
- Excessive or poorly mediated digital exposure may contribute to attentional fragmentation, sleep disruption, internalizing symptoms, and dysregulated patterns of use.

**What is the implication of the main finding?**

- Schools should move beyond a “handwriting versus digital tools” dichotomy and adopt developmentally sensitive hybrid models.
- Handwriting should be irreplaceable during key developmental stages, while digital tools should be used as purposeful accommodations rather than default replacements.

**Abstract:**

Background/Objectives: The digital transition in school settings is reshaping children’s learning, writing practices, and mental health trajectories. This narrative review examines whether handwriting still matters in contemporary hybrid educational environments from a multidisciplinary perspective. Methods: Evidence from neuroscience, developmental psychology, educational sciences, pediatrics, and child psychiatry was narratively synthesized, with attention to handwriting, digital exposure, learning, emotional regulation, and vulnerable populations. Results: Handwriting uniquely integrates fine motor control, visuomotor coordination, orthographic processing, attention, and embodied cognition, supporting early literacy, memory consolidation, conceptual learning, and reflective writing. Conversely, excessive or poorly mediated digital exposure may interact with attentional fragmentation, sleep disruption, online stressors, problematic use, and internalizing symptoms, particularly in vulnerable children and adolescents. Digital tools remain essential for personalization, accessibility, and compensatory support, especially for students with neurodevelopmental or learning difficulties. Conclusions: Handwriting and digital technologies should not be framed as competing educational paradigms. A developmentally sensitive hybrid model is needed, preserving handwriting during key stages of literacy and self-regulation while integrating digital tools as purposeful, individualized resources for learning, inclusion, and school mental health promotion.

## 1. Introduction

The rapid digitalization of childhood and school environments has changed the ways in which children learn, communicate, write, and relate to information. Digital devices are increasingly present in classrooms and may support personalization, accessibility, inclusion, collaborative learning, and assistive practices. At the same time, the growing use of screens, social media, and digital platforms has raised concerns about possible developmental and mental health consequences, particularly in relation to attention, sleep, emotional regulation, problematic use, and internalizing symptoms [1].

Within this broader transition, the role of handwriting in school education has become increasingly uncertain. Tablets, laptops, speech-to-text systems, and digital learning platforms are often perceived as faster, more efficient, and more consistent with contemporary educational demands. As a result, handwriting practice may appear less central than in the past. Handwriting is not simply an older way of producing written text. It is a complex developmental activity that involves fine motor control, visual–motor integration, graphomotor planning, attention, spatial organization, letter knowledge, spelling, and orthographic processing [2]. These components are closely related to handwriting development and written language acquisition, particularly because handwriting practice may strengthen visual representations of letters, support letter recognition, and contribute to spelling and orthographic learning. Broader oral language development and language acquisition follow partially independent developmental trajectories and should not be reduced to fine motor or graphomotor skills.

This issue is particularly relevant in childhood, when written language is still being consolidated. Handwriting, typing, and other writing modalities may not be developmentally equivalent, especially during early literacy acquisition. Handwriting practice may support letter learning, orthographic representations, spelling, and written language development, whereas typing and digital tools may offer advantages for speed, revision, accessibility, and compensatory support at later stages or in specific populations [3]. The central question, therefore, is not whether handwriting or digital writing is universally superior, but when, for whom, and for which learning goals each modality is most appropriate.

The mental health dimension adds further complexity. Digital technologies may offer social connection, educational support, access to information, and opportunities for self-expression. However, they may also contribute to attentional fragmentation, sleep disruption, social comparison, emotional dependence on online feedback, and problematic or addictive patterns of use in vulnerable children and adolescents [4,5]. These risks are unlikely to depend only on the amount of time spent on screens. Content, timing, context, emotional function, and the degree of dysregulation associated with digital engagement are also important [6].

The balance between handwriting and digital tools may be especially delicate in children with neurodevelopmental or psychiatric vulnerabilities. In ADHD, digital environments may interact with impulsivity, attentional control, and sleep regulation. In autism spectrum disorder, digital tools may support structured learning and communication, but they may also reinforce rigid or solitary patterns when poorly mediated. In dyslexia, dysgraphia, and other specific learning disorders, assistive technologies may reduce transcriptional barriers, while the premature removal of handwriting may limit opportunities for graphomotor and orthographic development [7,8,9,10].

A further distinction is needed between digital tools used to assess handwriting and computational approaches used to classify handwriting-related difficulties. Tablet-based handwriting assessment refers to the digital recording of handwriting performance through spatial, temporal, pressure-related, and kinematic parameters, such as stroke duration, velocity, pauses, pen pressure, fluency, and movement regularity. These methods have been used in handwriting research for many years and can provide objective information on handwriting quality and dynamics. Machine-learning approaches, by contrast, use handwriting-derived features to classify, predict, or detect handwriting difficulties. Although promising, these approaches should not be conflated with tablet-based assessment itself, because their clinical effectiveness in identifying handwriting impairments with clear educational or clinical relevance still requires stronger validation.

Addressing these questions requires a multidisciplinary perspective. Handwriting and digital tools cannot be understood only as educational techniques because they also involve motor development, cognitive processes, emotional regulation, inclusion, and mental health. Educational science, developmental psychology, neuroscience, child psychiatry, occupational therapy, and public health can therefore offer complementary perspectives on how writing modalities shape children’s learning and well-being.

A central premise of this review is that handwriting should be considered a specific developmental skill rather than a residual or merely traditional educational practice. Its acquisition depends on the progressive integration of fine motor control, visual–motor coordination, graphomotor planning, spatial organization, letter knowledge, spelling, and orthographic learning. These processes are particularly relevant during preschool and early primary education, when children are learning to associate visual letter forms with motor programs, phonological information, and emerging written language representations. Difficulties in handwriting, including dysgraphia, may therefore have consequences that extend beyond poor legibility, affecting writing fluency, cognitive load, academic participation, self-efficacy, and school inclusion. For this reason, the educational debate should not be reduced to a simple contrast between handwriting and digital tools but should address when handwriting instruction is developmentally necessary, when typing may become useful, and when assistive technologies are clinically or educationally indicated.

This narrative review examines whether handwriting still matters in the context of the digital transition of school settings. It integrates evidence on digital exposure and child mental health, the neurodevelopmental foundations of handwriting, the role of graphomotor activity in literacy and learning, the psychological meaning of writing by hand, emotional regulation, vulnerable populations, and educational and clinical implications. The aim is not to oppose handwriting and digital tools but to consider how both can be integrated as complementary resources for learning, inclusion, and mental health promotion.

## 2. Methods

### 2.1. Review Design

This article was designed as a narrative review adopting a multidisciplinary and developmentally informed perspective. The aim was not to answer a single, narrowly defined clinical question or to provide a quantitative estimate of effect size but to critically integrate evidence on the role of handwriting in the context of the digital transition of school settings. Particular attention was given to handwriting development, graphomotor skills, literacy acquisition, orthographic learning, handwriting difficulties, dysgraphia, digital writing modalities, and the educational and clinical implications of handwriting instruction in children and adolescents.

Given the breadth of the topic, the review integrates evidence from neuroscience, developmental psychology, educational sciences, occupational therapy, pediatrics, child psychiatry, and school mental health. The narrative approach was considered appropriate because the manuscript addresses a complex and interdisciplinary question involving heterogeneous study designs, populations, outcomes, and theoretical frameworks.

### 2.2. Search Strategy and Databases

A structured literature search was conducted in PubMed/MEDLINE, Scopus, Web of Science, and Google Scholar. The search covered articles published from January 2000 to March 2026, with the last search performed in March 2026. Earlier seminal studies were also considered when they provided foundational evidence on handwriting, literacy acquisition, graphomotor development, or the neurocognitive mechanisms of written language.

The search strategy combined terms related to handwriting and graphomotor development with terms related to literacy, digital technologies, neurodevelopment, learning, and mental health. The following search terms and combinations were used, adapted as appropriate for each database: “handwriting” OR “hand writing” OR “graphomotor” OR “graphomotor skills” OR “fine motor skills” OR “visual-motor integration” OR “visuomotor integration” OR “orthographic learning” OR “letter recognition” OR “literacy acquisition” OR “writing development” OR “handwriting fluency” OR “handwriting difficulties” OR “dysgraphia” OR “developmental dysgraphia” OR “handwriting assessment” OR “tablet-based handwriting assessment” OR “digital handwriting” OR “typing” OR “keyboard writing” OR “assistive technology” OR “digital education” OR “screen exposure” OR “school mental health”.

These terms were combined with population-related terms such as “children”, “adolescents”, “preschool”, “primary school”, “school-aged children”, “neurodevelopmental disorders”, “specific learning disorders”, “dyslexia”, “ADHD”, and “autism spectrum disorder”.

### 2.3. Inclusion and Exclusion Criteria

Studies were included if they met one or more of the following criteria: addressed handwriting development, graphomotor skills, fine motor prerequisites for handwriting, or visual-motor integration in children or adolescents; examined the relationship between handwriting, letter recognition, spelling, reading, orthographic learning, writing fluency, or written language development; compared handwriting with typing, keyboard writing, digital writing, or tablet-based writing; investigated handwriting difficulties, dysgraphia, handwriting assessment, or educational interventions targeting handwriting; addressed the use of typing, digital tools, or assistive technologies as compensatory strategies for children with handwriting, learning, or neurodevelopmental difficulties; provided relevant evidence on digital exposure, school digitalization, and child or adolescent mental health when directly relevant to the broader educational context of handwriting and digital transition.

Peer-reviewed empirical studies, longitudinal studies, randomized or quasi-experimental studies, neuroimaging studies, validation studies of handwriting assessment tools, systematic reviews, meta-analyses, and clinically or educationally relevant narrative reviews were prioritized.

Studies were excluded if they focused exclusively on adult populations without developmental relevance, addressed digital technology use without educational or developmental implications, examined mental health outcomes unrelated to school-age digital exposure or writing practices, or discussed writing only as a general linguistic process without reference to handwriting, graphomotor development, written production, or educational practice. Editorials, commentaries, conference abstracts, non-peer-reviewed sources, and studies with insufficient methodological detail were generally excluded, unless they provided important conceptual or historical context.

### 2.4. Source Selection and Prioritization Rationale

Because narrative reviews may be vulnerable to selection bias, source selection was guided by explicit criteria. Priority was given to studies that were directly relevant to the central focus of the review, namely the developmental, cognitive, educational, and clinical significance of handwriting in school-aged populations. Within each thematic domain, greater weight was assigned to systematic reviews, meta-analyses, longitudinal studies, experimental studies, validated assessment studies, and studies with clear developmental or educational implications.

For the sections on handwriting and literacy, priority was given to studies examining graphomotor development, handwriting fluency, letter recognition, spelling, reading acquisition, orthographic learning, and written composition. For the sections on handwriting difficulties and dysgraphia, priority was given to studies reporting prevalence data, assessment tools, and educational or clinical implications. For the sections on digital technologies, priority was given to studies that distinguished between educationally guided digital tool use, tablet-based handwriting assessment, typing, assistive technologies, and broader screen exposure.

Evidence on digital exposure and mental health was included selectively and only when it helped contextualize the school-based digital transition. These studies were not used to imply that handwriting directly prevents mental health problems, but rather to frame the broader developmental context in which decisions about handwriting, typing, and digital tools are made.

### 2.5. Thematic Integration of Evidence

The selected literature was synthesized thematically rather than quantitatively. Evidence was organized into the following conceptual domains: digitalization of school settings; neurodevelopmental foundations of handwriting; graphomotor development; handwriting, literacy, and orthographic learning; handwriting versus typing; handwriting difficulties and dysgraphia; compensatory digital tools and assistive technologies; emotional and self-regulatory dimensions of writing; and educational and clinical implications.

Findings from different disciplines were integrated according to developmental relevance, conceptual coherence, and strength of evidence. Neuroscientific studies were used to clarify the sensorimotor and neural mechanisms involved in handwriting; developmental and educational studies were used to interpret handwriting acquisition, literacy outcomes, and school-based instruction; occupational therapy and clinical literature were used to address handwriting difficulties, dysgraphia, and assessment tools; and child psychiatry and school mental health literature were used to contextualize digital exposure, emotional regulation, and vulnerable populations.

The synthesis aimed to distinguish well-supported evidence from more speculative hypotheses. Accordingly, conclusions were formulated cautiously, emphasizing that handwriting and digital tools should not be framed as mutually exclusive but as complementary resources whose use should be adapted to developmental stage, learning objectives, and individual needs.

### 2.6. Conceptual Distinctions Guiding the Review

Several conceptual distinctions guided the interpretation of the evidence. Tablet-based handwriting assessment and machine-learning approaches were considered related but distinct domains. Tablet-based handwriting assessment refers to the digital recording and quantitative analysis of handwriting performance, including spatial, temporal, pressure-related, and kinematic parameters such as stroke duration, velocity, pauses, pen pressure, fluency, and movement regularity. These approaches have been widely used in handwriting research and provide objective information on handwriting quality and dynamics. Machine-learning approaches, by contrast, involve computational models that use handwriting-derived features to classify, predict, or detect handwriting impairments. Although promising, these models should not be interpreted as clinically established diagnostic tools unless they are supported by external validation, normative data, interpretability, and evidence that they improve educational or clinical decision-making.

Handwriting instruction during literacy acquisition was distinguished from handwriting use in later educational stages. In preschool and early primary education, handwriting instruction contributes to graphomotor development, letter recognition, spelling, orthographic learning, and the progressive automatization of written production. In later primary and secondary education, once transcription skills are more consolidated, typing may offer advantages for speed, revision, accessibility, and academic productivity.

The compensatory use of typing and assistive technologies was interpreted separately from handwriting instruction. For children with persistent handwriting difficulties, dysgraphia, specific learning disorders, ADHD, autism spectrum disorder, or other neurodevelopmental conditions, handwriting may become excessively effortful and cognitively demanding. In these cases, typing, speech-to-text tools, word processors, and other assistive technologies may represent appropriate accommodations that reduce transcriptional barriers and support participation. However, these tools should be individualized and should not lead to the premature removal of handwriting practice when graphomotor and orthographic skills are still developing.

Finally, the review distinguishes fine motor skills involved in handwriting from broader language acquisition processes. Fine motor control, visual–motor integration, and graphomotor planning are important prerequisites for handwriting and may support letter production, orthographic learning, and written expression. However, oral language development, vocabulary, grammar, narrative competence, and broader language acquisition follow developmental trajectories that are not reducible to fine motor development. This distinction is important to avoid overstating the role of handwriting in language learning while still recognizing its specific contribution to written language acquisition.

## 3. Digitalization of School Settings: Opportunities and Developmental Concerns

The digital transition has progressively transformed school settings, making tablets, laptops, interactive platforms, online resources, and technology-mediated learning increasingly embedded in children’s daily educational experience. This transformation offers important opportunities for personalization, accessibility, and inclusion, particularly for students with heterogeneous developmental profiles or special educational needs [11,12]. At the same time, schools represent a crucial developmental environment in which cognitive, emotional, social, and sensorimotor skills are actively shaped.

For this reason, the impact of digitalization cannot be evaluated only in terms of educational efficiency or technological innovation. Digital tools may support learning and participation when they are intentionally integrated into structured pedagogical models, but early, pervasive, or poorly regulated screen exposure may also raise concerns regarding attention, executive functioning, emotional regulation, sleep, social interaction, and mental health trajectories [1,13,14]. A developmentally informed perspective is therefore needed to distinguish purposeful digital integration from excessive or passive exposure and to understand how digital technologies can coexist with embodied, interpersonal, and non-screen forms of learning.

### 3.1. Digital Learning as a Tool for Personalization and Inclusion

Digital technologies have become increasingly integrated into school environments and may offer meaningful opportunities for personalization, accessibility, and inclusion. When used intentionally, digital tools can support differentiated instruction, provide multimodal access to information, and allow students to learn at a more individualized pace. These features may be particularly relevant for children with heterogeneous developmental profiles, including those with neurodevelopmental disorders, specific learning disorders, sensory difficulties, or physical disabilities [11].

Digital learning may also reduce barriers to participation by offering visual supports, immediate feedback, assistive functions, and alternative ways to communicate or demonstrate knowledge. In this sense, technology should not be viewed only as a substitute for traditional teaching but as a potential resource for adapting educational environments to individual needs. Evidence from digital platforms used by families of children with disabilities suggests that online tools may facilitate information seeking, social support, and connection with services, although access, privacy, and quality of information remain important concerns [12].

In school settings, the impact of digital tools appears to depend strongly on the pedagogical context in which they are embedded. Technology-supported cooperative learning, for example, has been associated with improved peer relationships and reduced victimization and mental health problems among adolescents, suggesting that structured digital tools may support not only academic learning but also social and emotional outcomes [15]. Similarly, selected digital interventions may be useful for specific vulnerable groups when they are developmentally appropriate and clinically guided [16].

The potential benefits of digital education should not be confused with unrestricted screen exposure. Recent recommendations emphasize the need for purposeful, age-sensitive, and balanced use of digital technologies in educational settings, preserving non-screen activities and developmentally relevant experiences [11]. Therefore, the key issue is not whether digital tools should be present in schools, but how they can be integrated without displacing embodied, interpersonal, and sensorimotor forms of learning.

### 3.2. Early and Pervasive Screen Exposure in Educational Contexts

Early childhood represents a sensitive period for cognitive, linguistic, motor, and socioemotional development. In this phase, learning is strongly supported by direct sensorimotor exploration, face-to-face interaction, shared attention, play, movement, and adult scaffolding. The increasing availability of digital devices in family and educational contexts has therefore raised concern about the developmental consequences of early and pervasive screen exposure, particularly when screen-based activities displace embodied and interpersonal experiences.

Evidence suggests that both the duration and the context of screen exposure are relevant. In preschool children, screen use above one hour per day has been associated with a higher likelihood of behavioral problems, delayed developmental milestones, and poorer vocabulary acquisition compared with lower exposure levels [17]. At the same time, recent meta-analytic evidence indicates that a purely quantitative screen-time model is insufficient: program viewing, background television, age-inappropriate content, and caregiver screen use during routines appear to be associated with less favorable cognitive or psychosocial outcomes, whereas co-use with adults may support cognitive development [14].

These findings are particularly relevant for school and preschool settings, where digital tools may be introduced before children have fully consolidated attention control, language skills, fine motor abilities, and self-regulation. Although well-designed digital content can support selected learning goals, especially when mediated by adults, early screen-based learning should not replace activities such as handwriting, drawing, manipulation of physical objects, symbolic play, outdoor movement, and dialogic interaction. These offline experiences provide multimodal feedback and relational cues that are central to early neurodevelopment.

Neurodevelopmental findings also support caution. In preschool-aged children, higher screen-based media use has been associated with lower integrity of white matter tracts involved in language, executive function, and emergent literacy, although causal conclusions cannot be drawn from cross-sectional evidence [18]. Overall, early digital exposure in educational contexts should be guided by developmental principles, prioritizing age-appropriate content, adult mediation, limited passive exposure, and the preservation of non-screen learning experiences [19].

### 3.3. Cognitive Load, Attentional Fragmentation, and Executive Functioning

The integration of digital devices into children’s daily learning environments may also influence attention and executive functioning, particularly when digital activities involve frequent switching between stimuli, multitasking, rapid feedback, or competing sources of information. Executive functions, including working memory, inhibitory control, cognitive flexibility, and sustained attention, are still developing throughout childhood and adolescence and are essential for academic learning, emotional self-regulation, and goal-directed behavior.

Media multitasking appears especially relevant in this context. In adolescents, greater media multitasking has been associated with poorer performance on standardized academic tests, lower working memory capacity, and higher self-reported impulsivity, while not being equally related to more basic cognitive or motor abilities such as processing speed or manual dexterity [20]. These findings suggest that the association between digital multitasking and cognition may be relatively specific to executive processes rather than reflecting a general reduction in cognitive ability. However, because most evidence is correlational, it remains unclear whether media multitasking contributes to weaker executive control, whether adolescents with poorer self-regulation are more likely to multitask, or whether both directions coexist.

The relationship between screen media use and attention-related behaviors has also been examined in the Attention Deficit Hyperactivity Disorder (ADHD) literature. A systematic review covering four decades of research found a small but statistically significant association between screen media use and ADHD-related behaviors, including attention problems, hyperactivity, and impulsivity, while emphasizing the importance of individual susceptibility and the need for stronger causal evidence [21]. Similarly, a longitudinal cohort study found that frequent engagement in multiple digital media activities among adolescents was modestly associated with subsequent ADHD symptoms over a 24-month follow-up [22].

Highly fragmented or overstimulating digital environments may increase cognitive load and interfere with sustained attention, especially in younger children or in students with pre-existing attentional vulnerabilities. By contrast, structured digital activities with clear learning goals, limited distractions, adult guidance, and appropriate pacing may reduce unnecessary cognitive demands and support learning. Therefore, the educational use of digital technologies should be designed to protect attentional continuity and executive functioning, rather than promoting constant switching between competing stimuli [11].

### 3.4. Digital Device Use and Internalizing Symptoms in Children

Beyond cognitive and attentional outcomes, increasing attention has been directed toward the relationship between digital device use and internalizing symptoms in children and adolescents. Screen exposure has been associated with several dimensions of psychological vulnerability, including depressive symptoms, anxiety, emotional distress, lower well-being, and poorer quality of life. These associations should be interpreted cautiously, given the heterogeneity of age groups, screen content, device type, duration of exposure, and study design [1,13].

A systematic review of reviews found moderately strong evidence for an association between higher screen time and depressive symptoms in children and adolescents, while evidence for anxiety, behavioral problems, hyperactivity and inattention, poorer psychosocial health, lower well-being, and sleep outcomes was weaker [1]. More recently, a systematic review focused on adolescents reported that most included studies found associations between screen exposure and mental health outcomes, particularly for smartphone and social media use, while emphasizing the need to better characterize screen content, context, and patterns of interaction [13].

In younger children, the psychosocial impact of screen exposure appears to depend not only on duration but also on the context in which screens are used. In preschool-aged children, higher screen time has been associated with behavioral problems, delayed developmental milestones, and poorer vocabulary acquisition [17]. Similarly, meta-analytic evidence indicates that age-inappropriate content and caregiver screen use during daily routines are associated with less favorable psychosocial outcomes, whereas adult co-use may represent a more supportive context [14].

These observations are clinically relevant because emotional development in childhood relies strongly on contingent interpersonal interaction, shared attention, symbolic play, routines, and opportunities for self-regulation. Excessive or poorly mediated screen exposure may displace some of these experiences, while children with pre-existing emotional or self-regulatory difficulties may also be more likely to receive screens as a calming or distraction strategy [19]. Thus, digital device use should be considered a context-dependent factor that may interact with individual vulnerability, family environment, school climate, and developmental stage.

From a school mental health perspective, these data support balanced digital integration that preserves social interaction, emotional engagement, movement, and non-screen activities [11,19]. The aim is not to eliminate digital tools from educational settings but to prevent excessive, passive, or poorly mediated use from amplifying emotional vulnerability in children and adolescents [11].

## 4. Neurodevelopmental Foundations of Handwriting

To strengthen the handwriting-specific focus of the review, this section examines handwriting as a developmental graphomotor skill that contributes to early literacy and written language acquisition. Particular attention is given to the prerequisites of handwriting, including fine motor control, visual-motor integration, motor planning, spatial organization, and the progressive automatization of letter production.

Handwriting is a complex developmental skill that integrates fine motor control, visual–motor coordination, perceptual processing, attention, language, and learning-related cognitive functions. Its acquisition depends on the maturation of graphomotor abilities and on opportunities for practice within family and school environments [2,23,24,25,26]. Neuroimaging and developmental evidence further suggest that handwriting recruits distributed brain networks involved in motor planning, visual letter processing, orthographic representation, and fine movement coordination, while handwritten letter production may support the early specialization of reading-related neural circuits [27,28]. These findings provide the basis for considering handwriting not as a redundant skill in the digital era but as a developmentally relevant activity for literacy acquisition and symbolic learning, particularly during preschool and early primary education [3,29,30].

### 4.1. Handwriting as a Sensorimotor-Cognitive Activity

Handwriting is not a purely mechanical act but a complex sensorimotor-cognitive activity that requires the integration of fine motor control, visual perception, eye–hand coordination, proprioceptive feedback, motor planning, attention, working memory, and language-related processes. Fine motor skills have been broadly defined as small muscle movements requiring close eye–hand coordination and include manual dexterity, visual–motor integration, graphomotor skills, and the controlled manipulation of tools such as pencils. In this perspective, handwriting represents one of the most developmentally relevant expressions of fine motor activity in school settings.

The cognitive relevance of handwriting is supported by evidence linking fine motor skills with broader cognitive and academic domains. Experimental and developmental research suggests that fine motor skills and working memory may predict gains in decoding, indicating that motor processes can contribute to early reading-related learning [2]. Beyond literacy-specific outcomes, fine motor abilities have also been associated with cognitive processes such as mental imagery, supporting embodied and grounded cognition perspectives in which sensorimotor experience contributes to cognitive development and internal representation [23].

From a developmental standpoint, handwriting requires children to transform visual symbols into planned motor sequences, monitor the spatial and temporal features of the written trace, and adjust movement according to visual and proprioceptive feedback. This continuous integration of perception, action, and cognition distinguishes handwriting from more simplified forms of written production, such as keyboard typing, in which the motor act is less directly related to the visual structure of letters. Therefore, handwriting can be understood as an embodied learning activity in which the hand does not merely execute a linguistic output but participates in the construction and consolidation of symbolic representations [2,23]. Importantly, this relationship should be interpreted with developmental specificity. Fine motor and graphomotor skills are strongly implicated in handwriting acquisition, letter production, visual–motor integration, and the consolidation of orthographic representations. They may therefore support reading and spelling acquisition through their contribution to written language development. This does not imply that language acquisition as a whole depends on fine motor skills, since oral language, vocabulary, grammar, and narrative competence follow broader and partially independent developmental pathways.

### 4.2. Fine Motor Development and Graphomotor Integration

Fine motor development is a central component of school readiness and early academic participation. In early childhood, fine motor skills support everyday activities such as eating, dressing, drawing, manipulating objects, and using writing tools, while also contributing to the transition into formal schooling. These skills include partially distinct but interrelated domains such as manual dexterity, visual–motor integration, perceptual–motor coordination, and graphomotor control [24].

Graphomotor skills represent a specific subset of fine motor skills involving the use of a writing instrument. They require the integration of visual-spatial perception, orientation discrimination, motor control, and pencil manipulation and are considered important for later academic achievement and everyday school participation. Their development is not only determined by biological maturation but also by environmental opportunities. In typically developing children aged 3–7 years, home literacy experiences and educational approaches have been shown to explain a substantial proportion of variance in graphomotor skills, highlighting the role of family and school environments in shaping early writing-related abilities [25].

At the beginning of primary school, graphomotor performance becomes increasingly relevant because children are expected to use handwriting as a tool for learning rather than only as a skill to be acquired. In school beginners aged 6–8 years, fine motor and graphomotor performance have been shown to form a relatively distinct domain of motor functioning, separate from broader fitness and perceived motor competence, suggesting that fine motor skills deserve specific educational attention during the onset of handwriting instruction. This is particularly important because graphomotor tasks such as drawing, tracing, and writing require coordinated visual–motor processing, strength control, spatial organization, and cognitive monitoring [26].

Evidence from intervention research further supports the developmental significance of fine motor skills. A systematic review and meta-analysis of motor skill interventions in typically developing children from birth to 6 years found positive effects on fine motor, visual–motor, and manual dexterity outcomes, although the evidence should be interpreted cautiously because of methodological variability and risk of bias in several studies [24]. Overall, these findings suggest that graphomotor development is a modifiable developmental domain and that early educational environments can play an active role in supporting the foundations of handwriting.

### 4.3. Brain Networks Involved in Handwriting Versus Typing

**Figure 1:**
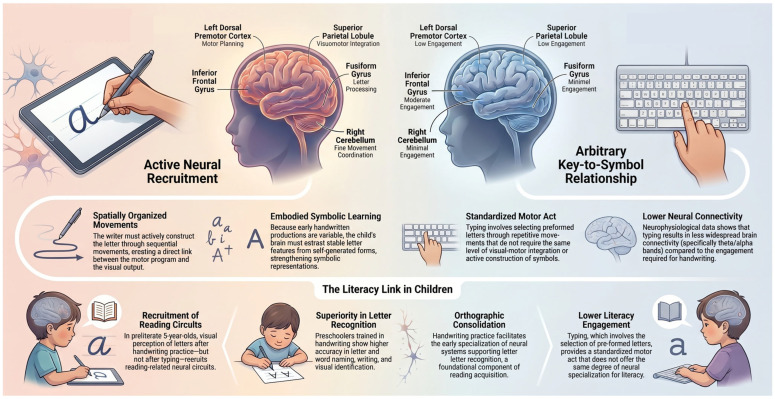
Neurocognitive pathways in handwriting vs. typing. Note. This figure illustrates the recruitment of distributed brain networks during letter production. Unlike typing, which relies on a standardized motor act to select a pre-existing symbol, handwriting involves active visuospatial construction. This process facilitates the early recruitment of reading-related neural circuits, specifically the fusiform gyrus and superior parietal lobule, strengthening the integration of visual, motor, and symbolic representations.

Neuroimaging evidence supports the view that handwriting is not simply a peripheral motor output but a complex brain-based activity involving coordinated linguistic, visual, motor, and sensorimotor systems. In middle childhood, fMRI data indicate that handwriting recruits a network already described in adults, including the left dorsal premotor cortex, superior parietal lobule, fusiform gyrus, inferior frontal gyrus, and right cerebellum. In children aged 8–11 years, this core network appears to be already established, although developmental differences remain: children show greater recruitment of prefrontal regions, suggesting higher cognitive control and less automatized execution, whereas adults show stronger activation in regions related to motor expertise and automatization, such as the right precentral gyrus and right anterior cerebellum [27]. The relationship between writing modality and the depth of cognitive encoding, particularly regarding the trade-off between transcription volume and conceptual understanding, is summarized in Figure 1.

The distinction between handwriting and typing is particularly relevant during early literacy acquisition. In preliterate five-year-old children, previous handwriting experience influenced brain activation during subsequent letter perception differently from typing or tracing. After children printed letters by hand, but not after they typed or traced them, visual perception of those letters recruited a reading-related circuit, including regions involved in letter processing and visual–motor integration. This suggests that self-generated handwriting may facilitate the early recruitment of neural systems supporting letter recognition and reading acquisition. This difference may depend on the specific sensorimotor properties of handwriting. Unlike typing, in which pressing a key has an arbitrary relationship with the visual form of a letter, handwriting requires the child to actively construct the letter through sequential and spatially organized movements. The visual output is directly linked to the motor program that produced it, and early handwritten productions are variable, requiring the child to extract stable letter features from self-generated forms. This may strengthen the coupling between visual letter representations and motor experience, supporting the development of more robust symbolic representations [28].

Additional neurophysiological evidence reinforces the complexity of handwriting as a learned fine motor skill. In an intracortical recording study of attempted handwriting, human motor cortex activity during complex Chinese character writing was organized as a sequence of stable neural states corresponding to stroke fragments, rather than as a simple continuous mapping of movement direction. Although this evidence is not developmental and does not directly compare handwriting with typing, it illustrates the hierarchical neural organization required for skilled handwriting [31].

All these findings suggest that handwriting and typing are not neurocognitively equivalent. Typing may efficiently support text production once literacy is established, but handwriting appears to provide a unique sensorimotor experience during the period in which children are learning to associate visual symbols, motor actions, and linguistic representations. From a developmental perspective, this supports caution against the premature replacement of handwriting with keyboard-based production in early schooling [27,28].

### 4.4. Handwriting, Neural Plasticity, and Early Literacy Acquisition

Handwriting may play a specific role in early literacy acquisition because it links visual symbols, motor actions, phonological information, and emerging orthographic representations. In the early stages of reading development, children must learn to recognize letters, associate them with sounds, reproduce them, and combine them into stable word forms. Fine motor skills and handwriting practice may support this process by strengthening the connection between perceptual, motor, and linguistic components of written language [2].

Behavioral and neurodevelopmental evidence suggests that the motor conditions of letter learning can influence subsequent literacy-related outcomes. In preschool children, handwriting practice has been associated with better letter recognition than typing, particularly in older preschoolers after repeated training [29]. Similarly, in preliterate children, printing letters by hand, but not typing or tracing them, was associated with the recruitment of reading-related brain regions during later letter perception, suggesting that self-generated handwriting may contribute to early neural specialization for letters [28].

Training studies further indicate that handwriting may support literacy acquisition beyond isolated letter recognition. In preschool children, pen-based handwriting training was superior to keyboard-based typing for word writing and showed a tendency toward better word reading, without any advantage of typing over handwriting [30]. More recently, prereading children trained through hand-copying or tracing showed higher accuracy in letter and word naming, writing, and visual identification than children trained through typing-based conditions, supporting the role of graphomotor experience in alphabetic and orthographic learning [3,30].

The advantage of handwriting may depend on the direct relationship between the visual form of a letter and the motor sequence required to produce it. Unlike typing, in which pressing a key is arbitrarily related to letter shape, handwriting provides visual, tactile, proprioceptive, and motor feedback while the child actively constructs the letter. This sensorimotor specificity may be particularly important when letter knowledge, decoding, spelling, and orthographic representations are still being consolidated [3,29,30].

Current evidence suggests that handwriting should not be considered redundant in the digital era. Although typing is important for later academic and communicative efficiency, handwriting may provide developmentally specific input during early literacy acquisition. Therefore, digital writing tools should be introduced in school settings without prematurely replacing handwriting practice, especially in preschool and early primary education [3,28,29,30].

## 5. Handwriting, Learning, and Cognitive Development

From an educational perspective, handwriting instruction is most relevant when it supports the transition from emergent graphomotor skills to fluent written language production. During this transition, handwriting practice may contribute to letter recognition, spelling, orthographic learning, and writing fluency, while persistent handwriting difficulties may increase cognitive load and interfere with higher-order writing processes. The neurodevelopmental foundations described above suggest that handwriting should not be considered only as a motor output but also as a learning-related activity. By integrating visual perception, fine motor control, attention, language, and orthographic processing, handwriting may support the consolidation of symbolic representations and the development of written language skills [2,3,28,29,30].

In school settings, the relevance of handwriting extends beyond early letter formation. As children progress through primary education, handwriting fluency and transcription skills may influence cognitive load, memory encoding, conceptual learning, spelling, and the quality of written composition [32,33,34,35]. At the same time, typing offers clear educational advantages, particularly for speed, accessibility, revision, and assistive purposes. Therefore, the question is not whether handwriting or typing should prevail, but how both modalities can be integrated according to developmental stage, learning goals, and individual needs [36,37,38,39,40,41,42].

### 5.1. Handwriting, Memory Consolidation, and Depth of Encoding

Handwriting may contribute to learning because it engages perceptual, motor, linguistic, and attentional processes involved in the active encoding of written symbols. As discussed above, writing letters by hand requires children to construct visual forms through sequential motor programs, proprioceptive feedback, and visual monitoring. This sensorimotor involvement may strengthen the association between letter shapes, motor patterns, phonological representations, and emerging orthographic knowledge, particularly during early literacy acquisition [2,3,28,30].

Unlike typing, in which preformed letters are selected through relatively arbitrary key presses, handwriting requires the learner to reconstruct each symbol through movement. This may promote deeper attention to the spatial structure of letters and words and support the stabilization of symbolic representations. Evidence showing advantages of handwriting practice over typing for letter recognition, word writing, and early literacy outcomes supports the idea that graphomotor experience may facilitate more durable learning-related representations [3,28,29,30].

The contribution of handwriting to learning also depends on fluency. When handwriting is slow or poorly automatized, low-level transcription processes may absorb cognitive resources that would otherwise support spelling, sentence generation, conceptual organization, and meaning construction. Conversely, more fluent handwriting may reduce cognitive load and allow children to allocate greater attention to higher-order aspects of written expression. Consistently, studies in primary school children indicate that handwriting fluency, spelling, and transcription skills are closely associated with writing quality and productivity [32,33,34,35].

Overall, handwriting may support learning through both sensorimotor encoding and progressive automatization. In early literacy, it helps link perception, action, and language; later, handwriting fluency may free cognitive resources for comprehension, memory integration, and written composition. Therefore, handwriting remains developmentally relevant in digitalized school environments, not as an alternative to typing but as a complementary pathway for consolidating symbolic learning and supporting written language development [2,3,28,29,30,31,32,33,34,35].

### 5.2. Handwriting Versus Typing in Academic Learning

The comparison between handwriting and typing is particularly relevant in contemporary school settings, where digital devices are increasingly used not only to access information but also to produce written work and take notes. Typing offers clear practical advantages, including speed, legibility, easy editing, and accessibility for students with specific motor or learning difficulties. Handwriting and typing may not support learning through identical cognitive mechanisms.

The developmental meaning of handwriting and typing changes across school stages. During preschool and the first years of primary education, explicit handwriting instruction may support graphomotor development, letter recognition, spelling, reading acquisition, orthographic learning, and the progressive automatization of written production. In this phase, handwriting provides a direct relationship between the visual form of letters and the motor sequences required to produce them, thereby strengthening visual and orthographic representations that are relevant for literacy acquisition [43].

Typing, by contrast, is generally not explicitly taught during the earliest years of primary school and relies on partially different cognitive and motor mechanisms. Although typing is faster and more efficient in many academic contexts, it requires the selection of preformed symbols through key presses that are more arbitrary in relation to letter shape. Experimental evidence suggests that handwriting and typing differ in the way linguistic and peripheral-motor processes interact during written production, with typing involving different mechanisms for segmenting, maintaining, and retrieving orthographic representations during motor execution [44]. Therefore, typing should not be interpreted as a developmentally equivalent substitute for handwriting during early literacy acquisition.

At later educational stages, however, typing may become increasingly useful for speed, revision, accessibility, and academic productivity. This distinction is particularly important for children with handwriting difficulties, dysgraphia, specific learning disorders, ADHD, autism spectrum disorder, or other neurodevelopmental vulnerabilities. In these children, handwriting may remain slow, effortful, or insufficiently automatized, absorbing cognitive resources that would otherwise support spelling, sentence generation, text organization, and revision. When handwriting imposes excessive cognitive or motor demands, typing and assistive technologies may represent appropriate compensatory strategies that reduce transcriptional burden and support academic participation [45].

A developmentally sensitive approach should therefore avoid both premature replacement of handwriting and rigid preservation of handwriting when it becomes a functional barrier. Handwriting should be explicitly supported during the period of graphomotor and literacy acquisition, while typing and assistive technologies should be progressively introduced according to age, task demands, functional impairment, and individual need [46].

Evidence from note-taking studies suggests that handwriting may promote more selective and elaborative processing, whereas typing may favor faster but more verbatim transcription. In children aged 10–11 years, handwritten and typed note-taking did not differ in factual recall, but children who took handwritten notes showed better conceptual understanding one week after the lesson [36]. This finding is consistent with adult research suggesting that longhand note-taking may encourage students to summarize and reorganize information rather than transcribe it mechanically [37].

**Figure 2:**
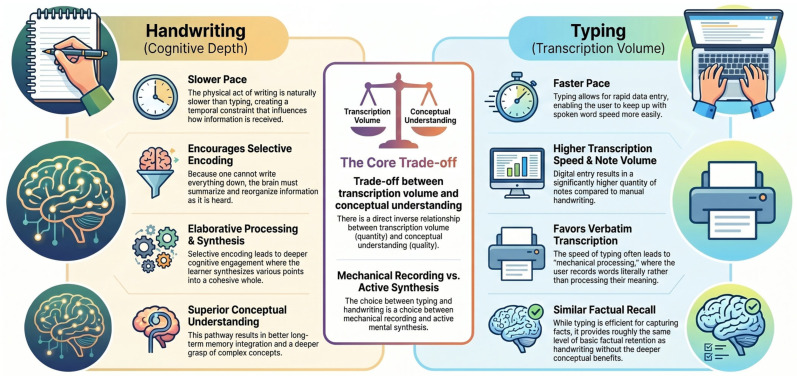
Cognitive implications of writing modality on learning outcomes. Note. The figure contrasts the effects of writing modality on encoding depth and memory. While typing enables higher transcription speed and note volume, it often leads to shallow, verbatim processing. Conversely, the slower pace of handwriting encourages selective encoding, information reorganization, and synthesis, promoting superior conceptual understanding and long-term memory integration.

The relationship between writing modality and the depth of cognitive encoding, particularly regarding the trade-off between transcription volume and conceptual understanding, is summarized in Figure 2.

Recent meta-analytic evidence in college students indicates that handwritten notes are associated with higher academic achievement, whereas typed notes are associated with greater note volume [38]. Although these findings cannot be directly generalized to younger children, they support the idea that the educational value of a writing modality depends not only on how much information is recorded but also on how actively it is processed. In school-aged children, this distinction may be especially important because note-taking, written production, and conceptual learning are still developing.

Neurophysiological evidence also suggests that handwriting and typing may involve different patterns of brain engagement. A high-density EEG study in young adults reported more widespread theta/alpha connectivity during handwriting than typewriting, a finding interpreted as potentially relevant for memory and learning [39]. However, this evidence should be interpreted cautiously because the study involved adults, did not include a behavioral learning task, and used a typing condition that may not fully reflect natural keyboard use [40].

Typing should not be considered cognitively inferior or educationally inappropriate, as it offers important advantages in terms of speed, accessibility, revision, and assistive support. However, handwriting may provide specific benefits for conceptual processing and learning retention, particularly when students are required to select, transform, and integrate information. A balanced educational approach should therefore preserve handwriting during key developmental stages while progressively introducing typing as a complementary tool according to learning goals and individual needs [36,37,38,39,40].

The evidence reviewed above suggests that handwriting, typing, and digital writing tools should not be considered interchangeable modalities. Rather, each engages partially distinct sensorimotor, cognitive, and educational processes, and each may be more or less appropriate depending on developmental stage, learning objectives, and individual vulnerability. To support this developmental interpretation, Table 1 provides a comparative synthesis of the potential strengths, limitations, and educational implications of handwriting, typing, and digital writing tools in school-aged children.

### 5.3. Implications for Reading, Spelling, and Written Language Development

Handwriting has important implications for reading, spelling, and written language development because it connects visual symbols with motor actions, phonological information, and orthographic representations. Evidence from preschool and early school-aged children suggests that handwriting practice may support letter recognition, word writing, and early literacy acquisition more effectively than typing-based practice, particularly when children are still consolidating the relationship between letter forms, sounds, and written words [3,28,29,30].

Beyond early letter learning, handwriting is also part of a broader set of transcription skills that contribute to written expression. In kindergarten children, handwriting and spelling were found to make specific contributions to written expression even after accounting for oral language, reading, cognitive skills, and child characteristics [41]. This suggests that graphomotor and orthographic abilities are not merely peripheral aspects of writing but may influence how effectively children transform ideas into written language.

As children progress through primary school, handwriting fluency becomes increasingly relevant for writing quality. When letter production is slow or effortful, children may have fewer cognitive resources available for sentence construction, text organization, and revision. Conversely, fluent handwriting may support more efficient written production and allow greater attention to meaning and composition. Consistently, studies in primary school children show associations between handwriting fluency, spelling, text quality, and writing productivity [32,33,34,35]. In a large study of nearly 5000 primary grade students, handwriting fluency was significantly associated with writing quality, supporting the idea that automaticity in transcription remains relevant even after the earliest stages of literacy acquisition [42].

Handwriting should be considered a developmental component of written language rather than a secondary motor skill. While typing becomes increasingly useful for speed, revision, accessibility, and assistive support, handwriting may provide specific benefits during the period in which reading, spelling, and written composition are still being consolidated. In digitalized school environments, this supports a balanced approach in which typing is progressively introduced without prematurely displacing handwriting practice during key stages of literacy development [41,42].

### 5.4. Handwriting Difficulties and Dysgraphia: Prevalence and Educational Relevance

Handwriting difficulties are relatively common among school-aged children, particularly during the early years of primary education, when graphomotor control, letter formation, writing speed, and handwriting fluency are still being consolidated. Prevalence estimates vary substantially across studies because of differences in age, language, educational context, assessment tools, and diagnostic thresholds. Nevertheless, available evidence indicates that handwriting difficulties are not rare and may represent a clinically and educationally relevant problem.

European data suggest that dysgraphic handwriting may be especially frequent during the first years of handwriting instruction and may decrease as handwriting becomes more automatized. In a study of Dutch primary-school children assessed with the Concise Assessment Scale for Children’s Handwriting, dysgraphic handwriting decreased from 37% to 17% in grade 2 and to 6% in grade 3, indicating that some early difficulties may improve with development and instruction, whereas persistent difficulties require closer monitoring and support. More broadly, handwriting difficulties have been estimated to affect approximately 10–34% of school-aged children, with higher rates reported in boys in several studies [47].

Italian evidence has also contributed to this field. The Italian validation of the Concise Assessment Scale for Children’s Handwriting evaluated 562 children aged 7–11 years from public primary schools and provided normative data for handwriting quality and copying speed in an Italian population. This study confirmed the usefulness of standardized handwriting assessment in Italian school-aged children and highlighted the influence of sex and school level on handwriting performance. Such data is important because handwriting norms may differ across countries as a result of language characteristics, educational practices, handwriting models, and curricular expectations [48].

The educational relevance of handwriting difficulties extends beyond poor legibility. When handwriting is slow, effortful, or poorly automatized, children may allocate excessive cognitive resources to transcription, leaving fewer resources available for spelling, sentence generation, text organization, revision, and conceptual expression. Persistent handwriting difficulties may therefore interfere with academic participation, written composition, self-efficacy, motivation, and school inclusion. In some children, handwriting difficulties may occur in association with developmental coordination disorder, specific learning disorders, ADHD, autism spectrum disorder, or other neurodevelopmental conditions.

For these reasons, early identification and standardized assessment are essential. Educational responses should be developmentally sensitive: children in the early stages of literacy acquisition should receive explicit handwriting and graphomotor instruction, whereas children with persistent dysgraphia or severe handwriting difficulties may require individualized accommodations, occupational therapy input, and compensatory tools such as typing or assistive technologies. The aim should not be to abandon handwriting prematurely but to prevent handwriting difficulties from becoming a barrier to learning, participation, and psychological well-being.

## 6. Embodied Cognition and the Psychological Meaning of Writing by Hand

The educational relevance of handwriting extends beyond literacy and academic performance. From an embodied cognition perspective, writing by hand can be understood as an activity in which perception, movement, attention, language, and material interaction are closely integrated. In this sense, handwriting is not only a way of recording already-formed thoughts but also a bodily mediated process through which children organize symbols, meanings, and self-related experiences [23,49,50,51].

### 6.1. Handwriting as an Embodied Cognitive Process

**Figure 3:**
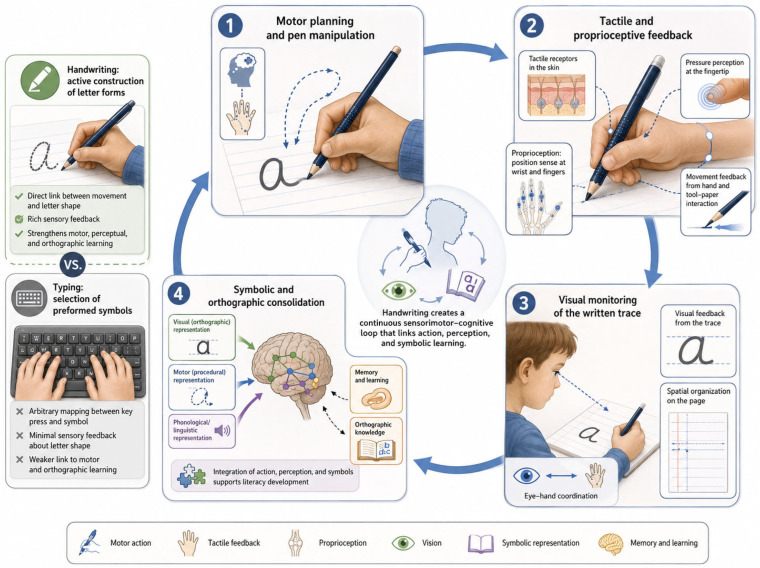
The sensorimotor-cognitive loop of handwriting as an embodied learning activity. Note. The figure illustrates handwriting as a dynamic loop involving motor planning, pen manipulation, tactile and proprioceptive feedback from the fingers and hand, visual monitoring of the written trace, and the consolidation of symbolic and orthographic representations. Unlike typing, handwriting creates a direct link between the physical construction of a letter and its symbolic representation, thereby supporting the integration of motor, visual, and cognitive processes involved in literacy and memory.

Embodied cognition theories emphasize that cognitive development is shaped by the body’s actions and interactions with the environment. In children, sensorimotor experiences contribute to the development of concepts, language processing, and symbolic understanding, suggesting that learning cannot be fully separated from bodily experience [49]. Handwriting represents a particularly relevant example of this principle because it requires children to coordinate visual perception, fine motor control, proprioceptive feedback, spatial organization, and linguistic representation within a single action. This integrated sensorimotor process can be conceptualized as a continuous dynamic loop, as illustrated in Figure 3.

In handwriting, the child does not merely select a preformed symbol but actively constructs it through movement. The graphic trace remains visible on the page as the product of the child’s own motor act, creating a direct link between action, perception, and symbolic form. This differs from typing, where the motor act is spatially and visually separated from the produced letter and where the relationship between movement and symbol is more arbitrary. The haptic and visuomotor properties of handwriting may therefore provide a distinctive form of embodied experience, particularly during the stages in which children are learning to associate letters, sounds, words, and meanings [3,28,29,30,50,51].

This embodied dimension also helps explain why handwriting may have educational value even in technologically advanced classrooms. Digital writing tools can support speed, accessibility, and revision, but they do not fully reproduce the sensorimotor contingencies of pen-on-paper writing. For this reason, handwriting may remain especially relevant in early and middle childhood, when symbolic learning, fine motor development, and cognitive control are still being consolidated [2,23,50,51].

### 6.2. Handwriting, Self-Reflection, and Psychological Meaning

Writing is also a psychological act through which experiences can be selected, organized, and transformed into language. Although most research on self-reflection, expressive writing, and narrative identity does not specifically compare handwriting with typing, it provides a useful framework for understanding why writing activities may have developmental and mental health relevance. Narrative identity research suggests that individuals progressively construct meaning by linking past experiences, present concerns, and future possibilities into coherent self-related narratives [52].

In school-aged children and adolescents, written activities may support this developmental process by offering a structured space for reflection, perspective-taking, and emotional articulation. Writing can help transform diffuse thoughts and feelings into ordered language, allowing children to externalize experience, create distance from it, and reorganize it in a more coherent form. In clinical and educational contexts, writing-based techniques have been described as tools that may support self-understanding, emotional processing, meaning-making, and psychological well-being [53].

The specific role of handwriting within these processes remains insufficiently studied. However, because handwriting is slower, bodily grounded, and materially traceable, it may encourage a more reflective pace than rapid digital production in some contexts. This does not imply that handwritten self-reflection is intrinsically superior to digital writing, but rather that the medium may shape the quality of attention, bodily engagement, and subjective experience involved in writing. In school settings, handwritten journals, reflective exercises, and narrative activities may therefore represent low-cost practices through which children can integrate cognitive, linguistic, and emotional dimensions of experience [53,54].

## 7. Handwriting, Emotional Regulation, and School Mental Health

Emotional regulation is a central developmental process in childhood and adolescence, with important implications for anxiety, depressive symptoms, stress vulnerability, and school functioning. In this context, writing-based practices may offer a structured way to transform internal experiences into language, supporting reflection, emotional awareness, and meaning-making. However, the available evidence mainly concerns expressive, reflective, or positive writing in general, rather than handwriting specifically. Therefore, handwriting should be considered here as a possible embodied modality within a broader family of writing-based practices, rather than as an independently established therapeutic mechanism [55,56,57].

### 7.1. Writing, Emotional Processing, and Self-Regulation

Writing may support emotional processing by helping children and adolescents organize affective experiences into coherent language. Expressive writing interventions are typically based on the idea that putting thoughts and emotions into words can facilitate cognitive reappraisal, emotional distancing, and narrative organization. These mechanisms may be particularly relevant during adolescence, a developmental period characterized by increased emotional reactivity, ongoing maturation of regulatory systems, and higher vulnerability to anxiety and depressive symptoms [57,58].

Recent evidence suggests that expressive writing may have small but potentially meaningful effects on psychological distress. A meta-analytic review of randomized studies with follow-up assessments found delayed and durable effects of expressive writing on depression, anxiety, and stress symptoms, although the magnitude of effects and the optimal conditions for intervention remain variable [56]. In school settings, a recent scoping review highlighted expressive writing as a flexible and low-cost approach, while also emphasizing substantial heterogeneity in implementation, reporting quality, and outcome assessment [55].

These findings support the potential relevance of writing-based activities for school mental health, but they should not be overinterpreted. Most studies do not isolate handwriting from other writing modalities, and many interventions focus on written emotional disclosure or positive writing rather than graphomotor processes. Nevertheless, from the perspective developed in the previous sections, handwriting may add an embodied and slower-paced component to reflective writing, potentially encouraging attentional engagement and self-monitoring. This hypothesis remains plausible but insufficiently tested, and future research should directly compare handwritten and digital writing in relation to emotional processing and regulation.

### 7.2. Handwriting-Based Activities as Low-Cost School Mental Health Practices

In educational contexts, writing-based activities may be particularly useful because they are inexpensive, scalable, and easily adaptable to classroom routines. Reflective journals, positive writing exercises, gratitude diaries, narrative prompts, and brief expressive writing tasks can be implemented without medicalizing emotional difficulties, while still offering students a structured space to name, organize, and reflect on internal experiences.

Evidence from positive writing interventions in adolescents supports this possibility. A resource diary intervention showed potential benefits for well-being and depressive vulnerability in adolescence, suggesting that writing about personal resources and positive experiences may help students identify coping capacities and supportive aspects of their lives [59]. More recently, an RCT conducted among schoolchildren aged 10–15 years during the COVID-19 period found that positive expressive writing reduced depressive and social anxiety symptoms compared with control conditions, supporting its potential as a low-resource intervention in school-aged populations [60]. Another recent study reported that positive psychology expressive writing influenced adolescents’ time attitudes and depressive symptoms, further suggesting that structured writing may help organize emotional experience across past, present, and future perspectives [61].

For the purposes of this review, these findings are relevant not because they demonstrate a specific clinical effect of handwriting, but because they show that structured writing can be used as a developmentally appropriate tool for emotional reflection and mental health promotion. Handwritten activities may be especially suitable for younger students or in contexts where the educational goal is not speed or productivity but reflective pacing, bodily engagement, and personal meaning-making. At the same time, digital writing may remain useful for accessibility, privacy, revision, or students with graphomotor difficulties.

Handwriting-based emotional activities should not be presented as substitutes for psychological or psychiatric care. Rather, they may represent low-cost, non-stigmatizing practices that can be integrated into broader school mental health promotion programs. In a balanced hybrid educational model, handwriting may contribute not only to literacy and learning but also to reflective routines that support emotional awareness, self-regulation, and psychological well-being [55,56,57,59,60,61].

## 8. Digital Exposure and Mental Health Risks in School-Aged Children

**Figure 4:**
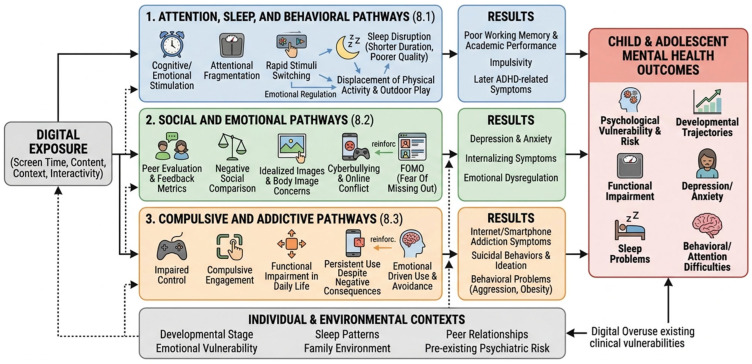
Mechanistic pathways linking digital exposure to mental health outcomes in children and adolescents. Note. The figure illustrates the three primary interacting pathways (behavioral, social, and addictive) through which digital media engagement may influence psychological vulnerability and developmental trajectories.

The relationship between digital exposure and child and adolescent mental health is complex and should not be reduced to a simple screen-time model. Digital technologies differ in content, context, timing, interactivity, social functions, and degree of problematic or compulsive use. Moreover, children and adolescents differ in developmental stage, emotional vulnerability, sleep patterns, family environment, peer relationships, and pre-existing psychiatric risk. For this reason, the mental health implications of digital exposure are better understood through multiple interacting pathways, including attentional and behavioral mechanisms, sleep and sedentary displacement, online social stressors, and problematic or addictive patterns of use [1,13,62,63]. The integration and interaction of these specific mechanisms are conceptually mapped out in Figure 4.

### 8.1. Attention, Sleep, and Behavioral Pathways

One pathway linking digital exposure to mental health involves attention, impulsivity, behavioral regulation, and sleep. In school-aged children, screen-based activities may increase cognitive and emotional stimulation, promote rapid switching between stimuli, and displace offline activities that support self-regulation, including physical activity, face-to-face interaction, outdoor play, and sleep routines. These mechanisms are particularly relevant because attention and executive functions are still developing throughout childhood and adolescence and because sleep and movement behaviors are strongly connected with emotional and cognitive functioning.

As discussed above, media multitasking and frequent digital media engagement have been associated with poorer working memory, academic performance, impulsivity, and later ADHD-related symptoms, although causality remains difficult to establish [20,21,22]. In early adolescents from the ABCD Study, greater screen time was associated with poorer mental health, more behavioral problems, worse academic performance, and poorer sleep, although the effect sizes were modest and socioeconomic factors showed stronger associations with several outcomes [63]. These results suggest that screen time may contribute to risk but also that it should be interpreted within broader developmental and environmental contexts.

Sleep represents a particularly important indirect pathway. A systematic review focused on digital media use and sleep in late adolescence and young adulthood found associations with shorter sleep duration and poorer sleep quality, especially for general screen use, mobile phone use, internet use, and social media use [64]. This pathway is clinically relevant because sleep, emotional regulation, and psychiatric vulnerability are closely interconnected during childhood and adolescence, and social media exposure may further interact with psychological distress, emotional dysregulation, and developmental sex-specific trajectories [65,66].

In youth populations, the 24 h movement behavior framework further emphasizes that screen exposure should be considered together with physical activity and sleep rather than in isolation. A systematic review of children and adolescents found that meeting recommendations for physical activity, recreational screen time, and sleep duration was generally associated with better mental health indicators, with evidence suggesting a dose–response gradient across the number of recommendations met [67]. A broader systematic review of movement behaviors similarly found that high physical activity combined with low sedentary behavior was associated with more favorable psychological and educational outcomes, while sleep often contributed to the most favorable behavioral profiles [68].

This pathway may be especially relevant for children with existing clinical vulnerabilities. Among youth with overweight or obesity receiving mental health services, screen time and physical activity patterns were examined as modifiable health behaviors in a population already at elevated psychiatric and cardiometabolic risk [69]. Although this study focused primarily on lifestyle and cardiovascular health, it highlights the importance of considering digital exposure within broader behavioral profiles, particularly in children and adolescents using mental health services.

Evidence suggests that digital exposure may affect mental health partly through behavioral displacement and dysregulation of daily routines. Excessive or poorly timed screen use may interfere with sleep, reduce physical activity, increase sedentary behavior, and contribute to attentional fragmentation. However, these associations are not deterministic: structured, purposeful, and developmentally appropriate digital use may coexist with healthy routines when embedded in balanced school and family environments [62,63,64,67,68,69].

### 8.2. Online Stressors, Social Comparison, and Internalizing Symptoms

A second pathway concerns the social and emotional features of online environments. Social media platforms expose adolescents to peer evaluation, social comparison, feedback metrics, idealized images, cyberbullying, exclusion, online conflict, and potentially distressing or self-referential content. These features may be especially relevant during adolescence, when sensitivity to peer approval, identity formation, body image concerns, and emotional reactivity are developmentally heightened.

Available evidence suggests that excessive or problematic social media use is associated with depressive symptoms, anxiety, sleep problems, and lower well-being, although the direction of causality remains difficult to establish and effect sizes are generally modest [4,70,71,72]. Importantly, the risk appears to depend not only on the amount of time spent online but also on the psychological quality and timing of use, including compulsivity, nighttime engagement, emotional dependence, social comparison, and impairment in daily functioning.

For the purposes of this review, these findings are relevant because they contextualize the broader digital transition of school-aged children and adolescents. They do not imply that digital technologies are intrinsically harmful or that handwriting directly protects against internalizing symptoms. Rather, they support the need for balanced educational routines that preserve sustained attention, sleep regularity, embodied learning, face-to-face interaction, and developmentally appropriate writing practices.

### 8.3. Digital Overuse and Developmental Vulnerability

In the context of this review, digital overuse is relevant primarily when it interferes with learning routines, attention, sleep, self-regulation, and school participation. The central issue is not simply time spent on screens, but whether digital engagement becomes rigid, poorly regulated, emotionally driven, or functionally disruptive. Many children and adolescents use digital devices for learning, communication, entertainment, and social connection without developing impairment. Risk appears to increase when digital use is associated with impaired control, compulsive engagement, distress when not using, sleep disruption, reduced offline activities, or difficulties in academic functioning [5,73,74].

This distinction is important for school settings because handwriting, reading, studying, and written composition require sustained attention, working memory, visuomotor coordination, and the ability to tolerate effortful, non-immediate tasks. Dysregulated digital use may therefore become relevant when it co-occurs with attentional difficulties, sleep deprivation, emotional distress, loneliness, or pre-existing learning and neurodevelopmental vulnerabilities [5,73,74,75]. In these cases, digital environments may amplify difficulties in task persistence, written productivity, and self-regulated learning, while vulnerable students may also rely on digital media for avoidance, reassurance, or mood regulation.

Digital overuse should be understood as a developmental vulnerability marker rather than as an isolated cause of psychopathology. From the perspective of handwriting and school learning, the most relevant clinical question is whether digital engagement supports educational participation or instead displaces activities that require graphomotor practice, sustained attention, face-to-face interaction, sleep regularity, and embodied learning. This perspective provides a bridge to vulnerable populations, including children with ADHD, autism spectrum disorder, specific learning disorders, dysgraphia, anxiety, and mood symptoms, for whom the balance between handwriting instruction, digital tools, and compensatory technologies must be individualized.

## 9. Vulnerable Populations: Neurodevelopmental and Psychiatric Perspectives

Children and adolescents with neurodevelopmental or psychiatric vulnerabilities may be particularly sensitive to the balance between digital tools and handwriting-based activities. In these populations, digital technologies can provide meaningful accommodations, compensatory strategies, and access to learning; however, they may also amplify pre-existing difficulties in attention, sleep, emotional regulation, social interaction, or written expression [7,8,10,76,77]. A clinically informed perspective is therefore needed to distinguish adaptive digital support from premature substitution of embodied, graphomotor, relational, and self-regulatory learning experiences [9,76,77,78].

### 9.1. Children with ADHD: Attention, Impulsivity, and Digital Overstimulation

Children with ADHD may be especially vulnerable to the attentional and regulatory demands of digital environments. ADHD is characterized by difficulties in sustained attention, inhibitory control, impulsivity, reward sensitivity, and executive functioning, all of which may interact with highly stimulating digital media. Previous evidence already discussed in this review suggests associations between screen media use, ADHD-related behaviors, and later ADHD symptoms, although these relationships are generally modest and bidirectional [21,22].

Recent longitudinal evidence supports a more nuanced interpretation. A systematic review of longitudinal studies found reciprocal associations between digital media use and ADHD symptoms in children and adolescents, with more consistent associations for problematic digital media use than for screen time alone. This suggests both selection effects, whereby children with ADHD symptoms may be more likely to engage in dysregulated digital use, and media effects, whereby digital media may contribute to later attentional difficulties, possibly through reward-driven engagement, sleep disruption, or social mechanisms [7].

Mechanistic data further indicate that impulsivity may play a central role. In a five-year population-based adolescent cohort, screen time was associated with growth in ADHD symptoms, and impulsivity emerged as the most robust mediator of this association; social media use also showed lagged within-person effects mediated by impulsivity and response inhibition [79]. Sleep represents another relevant pathway: in a nationally representative sample, children with ADHD had higher odds of sleep insufficiency and bedtime irregularity, and increased screen time was among the factors associated with poorer sleep outcomes in ADHD [80].

At the same time, handwriting and graphomotor difficulties are common in ADHD and should not be overlooked. Dysgraphia has been reported as highly prevalent in this population, and a psychomotor handwriting intervention showed promising effects on handwriting quality in children with ADHD, including those with co-occurring developmental coordination disorder [78]. Therefore, in children with ADHD, educational planning should address both digital regulation and handwriting support: digital tools may reduce frustration and facilitate access to learning, but structured handwriting and psychomotor activities may still be relevant for graphomotor development, attention, and self-regulation [78,81].

### 9.2. Children with Autism Spectrum Disorder: Digital Tools Between Support and Rigidity

In children with autism spectrum disorder (ASD), digital technologies may offer important opportunities for communication, learning, emotional recognition, and social inclusion. Visual structure, predictability, immediate feedback, and individualized pacing can be particularly useful for children with ASD, especially when digital tools are embedded within clinically or educationally guided interventions. For example, a randomized clinical trial of an artificial intelligence-driven wearable digital intervention showed improvements in socialization in children with ASD receiving standard behavioral care, supporting the potential of digital tools as adjunctive supports for social learning [82]. Similarly, digital interventions have been explored in preschool children with ASD as accessible and scalable resources when they are developmentally appropriate and carefully supervised [16].

The use of digital tools in ASD also requires caution. Children with ASD may show restricted interests, repetitive behaviors, sensory sensitivities, social communication difficulties, and a preference for predictable environments, all of which may interact with digital media use [10].

Evidence on screen time and ASD remains complex. A systematic review and meta-analysis found an overall positive association between screen time and ASD, particularly in studies of general screen use among children, but this association became non-significant after adjustment for publication bias, and most included studies were observational. Therefore, these findings should not be interpreted as evidence that screen exposure causes ASD. Rather, they highlight the need to evaluate timing, content, developmental context, and bidirectional relationships [10].

For children with ASD, digital tools should therefore be used as structured supports rather than as unregulated substitutes for interpersonal, sensorimotor, and communicative experiences. The goal is not to avoid technology but to integrate it within individualized educational plans that preserve face-to-face interaction, play, handwriting or drawing when appropriate, and embodied learning activities [10,16,82].

### 9.3. Specific Learning Disorders, Dysgraphia, and Graphomotor Difficulties

Specific learning disorders and dysgraphia are directly relevant to the central question of this review. Children with dyslexia frequently experience writing difficulties because reading and writing share underlying linguistic, phonological, orthographic, and executive processes. Written expression depends on transcription skills, working memory, executive function, and text generation; difficulties in any of these domains may reduce spelling accuracy, handwriting fluency, text organization, and overall writing quality [8]. This is consistent with previous sections showing that handwriting fluency, spelling, and transcription skills are closely related to written composition in primary school children [32,33,34,35,41,42].

Dysgraphia is not a homogeneous condition. In a study comparing children with dysgraphia, children with developmental coordination disorder, and typically developing controls, both clinical groups showed poorer handwriting performance than controls, with individual differences in the type and severity of handwriting impairments. These findings support the need for detailed assessment rather than generic recommendations to “use a computer” whenever handwriting is difficult [9].

### 9.4. Anxiety and Mood Disorders: Digital Avoidance, Compensation, and Risk Amplification

Children and adolescents with anxiety or mood symptoms may also represent a vulnerable group in relation to digital exposure. Digital media can offer social connection, distraction, psychoeducation, and access to support, but it may also reinforce avoidance, reassurance seeking, nighttime arousal, social comparison, and emotional dependence on online feedback [4,70,71,72,83,84]. This is particularly relevant because depression and suicidality in adolescence are associated with multiple risk factors, including hopelessness, bullying, comorbid psychiatric disorders, adverse childhood experiences, substance use, and nonsuicidal self-injury [75].

In adolescents, social media use may interact with sleep and emotional well-being. A study of Scottish adolescents found that overall social media use, nighttime-specific use, and emotional investment in social media were associated with poorer sleep quality, lower self-esteem, and higher anxiety and depressive symptoms; nighttime-specific use predicted poorer sleep quality even after controlling for anxiety, depression, and self-esteem [83]. A systematic review on the interplay between social media use, sleep quality, and mental health in youth similarly emphasized that sleep may be an important mechanism linking social media use with psychological distress [84].

These observations are consistent with the broader evidence reviewed above showing associations between social media use, problematic social media use, depression, anxiety, sleep problems, and lower well-being [4,70,71,72]. It remains important to distinguish general use from clinically relevant patterns. For some adolescents, online spaces may provide connection and help-seeking opportunities; for others, especially those with anxiety, depression, low self-esteem, or social vulnerability, digital engagement may become a maladaptive strategy for avoidance or mood regulation [70,71,83,84].

In school settings, warning signs may include increasing nighttime use, reduced sleep, social withdrawal, irritability, declining academic functioning, excessive reassurance seeking, or distress when disconnected. Digital guidance for emotionally vulnerable students should therefore focus not only on limiting time but also on sleep hygiene, emotional regulation, online content, peer dynamics, and the function that digital use serves for the individual child [4,70,75,83,84].

### 9.5. Digital Tools as Accommodation Rather than Replacement

Across vulnerable populations, digital tools are most useful when they function as individualized accommodations rather than universal replacements for handwriting, embodied learning, or interpersonal interaction. Assistive technologies can reduce barriers, support inclusion, and improve participation for students with special educational needs, particularly when transcription demands interfere with the expression of ideas [76,77]. In children with communication and writing difficulties, speech-to-text technology has been shown to improve the quantity and quality of written production and self-esteem, supporting its feasibility as a compensatory tool for students who struggle with handwriting or written expression [76]. Effective implementation requires availability, teacher training, and careful matching between the selected technology and the child’s specific functional needs [77].

The key clinical and educational principle is personalization. For some children, typing, word processing, speech-to-text, or other digital scaffolds may be essential to access the curriculum and demonstrate knowledge. For others, premature replacement of handwriting may reduce opportunities to strengthen graphomotor fluency, fine motor control, orthographic learning, and embodied engagement with written language. This distinction is especially relevant because handwriting difficulties are heterogeneous and require individualized assessment rather than a single compensatory response [9]. Moreover, transcription skills, handwriting fluency, and spelling remain closely linked to written expression and writing quality during development [41,42].

A balanced approach avoids both technological rejection and technological substitution. Digital tools should support autonomy, inclusion, and participation while preserving developmentally meaningful offline activities, including handwriting practice when appropriate, drawing, manipulation of physical materials, movement, play, and face-to-face communication. In this perspective, technology should not be used to remove developmental challenges indiscriminately but to reduce disabling barriers while maintaining opportunities for skill acquisition. This provides the basis for an integrated model in which handwriting and digital tools are considered complementary resources rather than competing educational paradigms [3,42,76,77], while also acknowledging that the use of digital tools in vulnerable adolescents should be considered in light of evidence on sleep disruption, social comparison, emotional distress, anxiety and depressive symptoms, and problematic digital engagement [4,70,71,72,83,84].

The balance between handwriting and digital tools becomes particularly relevant when developmental stage and individual vulnerability are considered. As reviewed above, digital technologies may provide important compensatory and inclusive resources, especially for students with neurodevelopmental or learning difficulties, but they may also amplify attentional, emotional, sleep-related, or relational vulnerabilities when used excessively or without adequate mediation. Conversely, handwriting-based activities may support graphomotor development, literacy acquisition, reflective pacing, and emotional self-regulation, although they should not be imposed rigidly when they represent a functional barrier [85,86,87,88,89]. Table 2 summarizes developmentally informed clinical and educational recommendations for integrating handwriting and digital tools across different age groups and vulnerable populations.

## 10. Toward an Integrated Model: Digital Tools and Handwriting as Complementary Resources

The evidence reviewed so far suggests that the relationship between handwriting, digital tools, learning, and mental health cannot be adequately understood through a simple opposition between paper-based and screen-based practices. Handwriting, typing, and assistive technologies are different modalities of written expression, each with specific developmental, cognitive, educational, and clinical implications. An integrated model should therefore consider not only the medium itself but also the child’s age, neurodevelopmental profile, learning goals, emotional functioning, and broader school context [2,85].

### 10.1. Moving Beyond the Handwriting-Versus-Typing Dichotomy

A rigid contrast between handwriting and typing risks oversimplifying the developmental role of writing. Handwriting is not merely a slower version of typing: it involves fine motor control, visuomotor integration, proprioceptive feedback, orthographic processing, and embodied interaction with written symbols [2]. These features may be especially relevant in early literacy acquisition, when children are still consolidating letter forms, sound-symbol correspondences, spelling, and word learning [3].

At the same time, typing and digital writing tools have clear educational value. Keyboarding may support speed, editing, revision, accessibility, and participation, particularly in older students or in children with graphomotor, spelling, or written expression difficulties. More broadly, writing quality depends on multiple component skills, including transcription, spelling, executive functions, language, and text generation, whose relative contribution changes across development [85,86]. Therefore, the relevant question is not whether handwriting or typing is globally superior, but which modality best supports a specific developmental task at a specific time.

Recent work also suggests that keyboarding should not be treated as an automatic or effortless substitute for handwriting. Like handwriting, keyboarding requires practice, automaticity, and positive attitudes toward writing; young students may benefit from explicit support in developing both handwriting and keyboarding fluency [87]. In this perspective, handwriting and typing should be understood as partially distinct but complementary transcription modalities. Both can support written language development when introduced at appropriate developmental stages and matched to the child’s functional needs.

### 10.2. A Developmentally Sensitive and Clinically Informed Framework

A developmentally sensitive model should consider at least three dimensions: the child’s stage of literacy development, the function of the writing activity, and the presence of neurodevelopmental or psychiatric vulnerabilities. In early childhood and primary school, handwriting may have particular relevance for letter learning, orthographic representations, fine motor development, and the consolidation of written language skills [2,3]. In later stages, typing and digital tools may become increasingly useful for drafting, revision, accessibility, and academic productivity, especially when handwriting is no longer the primary learning target [85,87].

Clinical vulnerability further modifies this balance. In children with ADHD, digital media may interact with impulsivity, sleep problems, and attentional dysregulation, while handwriting difficulties may also require targeted psychomotor or graphomotor support [7,78]. In children with ASD, digital tools may provide structured and motivating support for communication and social learning, but unregulated screen exposure should still be interpreted cautiously and within the child’s developmental context [10,82]. In children with dyslexia, specific learning disorders, or dysgraphia, assistive technologies may reduce transcriptional barriers, but handwriting difficulties remain heterogeneous and require individualized assessment rather than automatic substitution [8,9].

The same individualized logic applies to emotional and psychiatric vulnerability. For adolescents with anxiety or depressive symptoms, digital environments may provide connection and support but may also reinforce social comparison, avoidance, nighttime arousal, sleep disruption, and emotional dependence on online feedback [83,84]. Therefore, the educational use of digital tools should be informed not only by academic performance but also by sleep, attention, emotional regulation, peer functioning, and the subjective function that digital engagement has for the student.

### 10.3. Principles for Balanced Integration in School Settings

In school settings, a balanced model should preserve handwriting during developmental periods in which graphomotor practice contributes to literacy, spelling, and written language consolidation while progressively introducing typing and digital writing tools as additional competencies rather than as replacements by default [3,42]. This is particularly important because premature elimination of handwriting may reduce opportunities to strengthen graphomotor fluency and embodied engagement with written language, whereas delayed access to digital accommodations may unnecessarily restrict participation in children with significant writing difficulties [9,76].

Assistive technologies should therefore be framed as accommodations that reduce disabling barriers and enhance access to the curriculum. Speech-to-text technology, for example, may support students with learning or writing difficulties by allowing them to express ideas despite transcriptional limitations, but its effectiveness depends on training, implementation context, and appropriate matching to the learner’s needs [76,89]. Similarly, teachers’ beliefs, professional preparation, and availability of assistive tools influence whether technology is used as a meaningful support or remains underused in inclusive settings [77].

Overall, digital tools and handwriting should be considered complementary resources within a flexible educational ecology. Handwriting remains developmentally meaningful for embodied learning, literacy acquisition, and graphomotor development, while digital tools can support accessibility, productivity, revision, and individualized accommodations [2,76]. The challenge for schools is not to choose one modality over the other but to define when, for whom, and for what purpose each modality should be used. This integrated perspective provides the basis for translating the evidence into educational and clinical recommendations.

## 11. Educational and Clinical Implications

The evidence reviewed in this article suggests that schools and clinicians should move beyond generic recommendations about screen time or handwriting practice. Educational and clinical decisions should instead consider the child’s developmental stage, learning needs, neurodevelopmental profile, emotional functioning, family context, and the quality and purpose of digital use. Recent pediatric guidance similarly emphasizes that children’s digital media experiences should be understood within a broader digital ecosystem, rather than only through the number of hours spent on screens [6].

### 11.1. Implications for Teachers and School Systems

For teachers and school systems, the main implication is that handwriting should not be prematurely removed from the curriculum, especially during the phases in which graphomotor practice may support letter learning, spelling, and written language development [2,3,42]. At the same time, typing and digital writing tools should be progressively introduced as additional skills, particularly when students are expected to draft, revise, organize, and produce longer written texts [85,87].

A balanced school approach should therefore combine handwriting, typing, and assistive technologies according to developmental stage and functional need. In early primary school, handwriting practice may remain important for consolidating sensorimotor and orthographic representations; in later years, digital tools may support writing fluency, revision, and academic participation. For students with specific writing difficulties, accommodations such as word processing or speech-to-text should be introduced without framing them as a failure of handwriting but as tools to reduce barriers and support access to the curriculum [76,89].

Teachers also need training to identify when technology is being used as an educational support and when it is becoming a source of distraction, avoidance, sleep disruption, or emotional dependence. The implementation of assistive technology depends not only on the availability of devices but also on teacher confidence, professional preparation, and appropriate matching between tools and individual needs [77].

### 11.2. Implications for Child Mental Health Professionals

For child psychiatrists, psychologists, pediatricians, and other mental health professionals, digital exposure should be explored as part of routine developmental and psychosocial assessment. The relevant question is not only “how much screen time?” but also what the child is doing online, when the use occurs, whether it displaces sleep or offline activities, and whether it is linked to avoidance, reassurance seeking, social comparison, dysregulated mood, or impaired functioning [4,5,6].

Clinicians should also ask about writing and school participation. Avoidance of written tasks, excessive fatigue during handwriting, slow written production, declining academic self-esteem, or reliance on digital tools without a clear educational plan may indicate graphomotor, attentional, or learning difficulties. In such cases, referral for school-based assessment, occupational therapy, neuropsychological evaluation, or specific learning disorder assessment may be appropriate [8,9,88].

This clinical approach is particularly relevant for vulnerable populations. In children with ADHD, digital media may interact with impulsivity, sleep problems, and attentional dysregulation, while handwriting difficulties may require targeted psychomotor support [7,78]. In children with ASD, digital tools may support structured learning and social communication, but their use should remain guided and developmentally appropriate [10,82]. In adolescents with anxiety or depressive symptoms, digital use should be evaluated in relation to sleep, social comparison, avoidance, peer stressors, and emotional regulation [4,70,71,72,83,84].

### 11.3. Screening and Early Identification of Graphomotor, Attentional, and Emotional Difficulties

Early identification is essential because handwriting difficulties, attentional dysregulation, and emotional symptoms can reinforce each other in school contexts. A child who writes slowly or illegibly may avoid written tasks, produce shorter texts, experience frustration, or appear inattentive; conversely, children with ADHD or anxiety may show handwriting difficulties secondary to impulsivity, poor planning, reduced persistence, or avoidance [7,9,78].

Screening before formal literacy instruction should focus on graphomotor and visual-motor prerequisites for handwriting rather than on handwriting performance itself. In preschool children, relevant domains include visual–motor integration, copying of simple geometric forms, spatial organization, pencil grasp, pressure modulation, line control, movement fluency, eye-hand coordination, and the ability to reproduce non-verbal graphic patterns. These prerequisites are particularly important because they can be assessed before children have acquired formal reading and writing skills and may help identify children who require closer monitoring or early support.

Assessment tools used at this stage should ideally be developmentally appropriate, minimally language-dependent, and applicable across different cultural and linguistic contexts. When teachers are supervised by healthcare professionals, the Beery–Buktenica Developmental Test of Visual-Motor Integration may be used to assess visual–motor integration and related non-verbal prerequisites for handwriting [90]. For school-based screening conducted by teachers, particularly in Italian and Swiss-German educational contexts and across different school grades, the GRAFOS-2 screening instrument may also be considered [91,92]. These tools do not replace a comprehensive clinical evaluation, but they may support early identification, referral decisions, and targeted educational planning.

Once formal handwriting instruction has begun, assessment should include tools that evaluate the quality, speed, fluency, and functional impact of handwriting production. Analytic paper-and-pencil tests, such as the Graph-motor and posture disorders of handwriting test (DGM-P) [93] and the Concise Assessment Scale for Children’s Handwriting, may be useful to examine specific components of handwriting performance, including legibility, letter formation, spatial organization, copying speed, and graphomotor efficiency. The Concise Assessment Scale for Children’s Handwriting has also been validated in an Italian primary-school population and provides normative data for handwriting quality and copying speed [48].

In addition to analytic tools, global or holistic scales may help evaluate handwriting legibility in a more functional way. The Handwriting Legibility Scale is a non-language-dependent scale designed to assess global legibility using free-writing samples and has shown reliability and validity in school-aged children [94]. Such instruments may complement more detailed analytic tests by capturing the overall readability and functional adequacy of handwriting in educational contexts. Therefore, assessment should not rely on a single score but should combine standardized handwriting measures, clinical or educational observation, and information on the child’s classroom participation, effort, and need for accommodations.

Screening should include both product and process indicators of handwriting. Teachers and clinicians should consider legibility, speed, letter formation, spacing, fatigue, pain, avoidance, and the child’s emotional reaction to writing. A systematic review of handwriting assessment tools emphasizes that legibility and speed assessments have an important role in identifying and evaluating handwriting problems in children, although psychometric quality and clinical utility vary across instruments [88]. This supports the use of structured, developmentally appropriate assessments rather than informal impressions alone.

Digital assessment may also contribute to early identification by capturing dynamic handwriting features such as pressure, pauses, speed, and movement patterns [95]. However, assessment should remain functional and educationally meaningful: the aim is not simply to label dysgraphia but to understand how handwriting affects learning, participation, self-esteem, and access to written expression [8,9].

### 11.4. Handwriting-Based and Hybrid Activities for School Mental Health Promotion

Handwriting-based activities may also have a role in school mental health promotion, especially when used as reflective and educational practices: journaling, narrative writing, gratitude exercises, resource diaries, and brief reflective prompts may help students organize thoughts and emotions, support self-reflection, and develop emotional vocabulary [55,59,60].

A hybrid model is therefore preferable. Handwriting can be used for early literacy, graphomotor development, reflective activities, and embodied engagement with written language, while digital tools can support revision, accessibility, assistive communication, and participation. Schools should avoid both universal handwriting replacement and rigid handwriting insistence. The most appropriate approach is individualized, developmentally sensitive, and coordinated across teachers, families, and clinicians [6,88].

The evidence reviewed in this article supports a shift from a dichotomous view of handwriting and digital technologies toward an integrated developmental framework. In this model, the impact of writing modalities and digital exposure on children’s learning and mental health depends on the interaction between developmental stage, educational context, individual vulnerability, adult mediation, and the functional purpose of technology use.

## 12. Future Directions Beyond Comparisons Between Handwriting and Digital Tools

Future research should move beyond broad comparisons between handwriting and digital tools and address more specific developmental, educational, and clinical questions. Longitudinal and experimental studies are needed to clarify how graphomotor development, handwriting fluency, letter recognition, spelling, reading, orthographic learning, written composition, and cognitive load interact across preschool and primary school years [2,3,28,29,30,31,32,33,34,35,41,42]. Such studies should distinguish transient immaturity in handwriting from persistent handwriting difficulties and should examine how early graphomotor performance predicts later written language outcomes.

A first priority is to better define when handwriting provides specific developmental benefits and when digital tools offer functional advantages. During early literacy acquisition, handwriting may support the integration of visual letter forms, motor programs, phonological information, and orthographic representations [3,28,29,30]. At later educational stages, once transcription skills are more consolidated, typing and digital writing tools may offer advantages for speed, revision, accessibility, and academic productivity [36,37,38,39,40,85,86]. Future studies should therefore compare handwriting, keyboarding, speech-to-text, and other assistive technologies according to developmental stage, task demands, and student characteristics.

Further research is also needed on handwriting difficulties, dysgraphia, and specific learning disorders. In children with dysgraphia, dyslexia, ADHD, ASD, or other neurodevelopmental vulnerabilities, handwriting may become excessively effortful and may interfere with written expression, academic participation, and self-efficacy [8,9,10,76,77,78]. Future studies should identify which children benefit most from targeted handwriting remediation, which children require compensatory tools, and how remediation and accommodation can be combined without prematurely eliminating opportunities for graphomotor development [3,8,9,42,76,77].

A second priority concerns handwriting assessment. Standardized paper-and-pencil tools remain essential for evaluating handwriting quality, legibility, speed, and functional impact. At the same time, tablet-based handwriting assessment may provide additional spatial, temporal, pressure-related, and kinematic information, allowing a more detailed characterization of handwriting performance [95]. However, tablet-based assessment should be distinguished from machine-learning classification. Machine-learning approaches may support future screening or prediction of dysgraphia and other handwriting-related difficulties, but their educational and clinical value depends on external validation, interpretability, normative data, and evidence that they improve decision-making in real school and clinical contexts [95,96,97,98,99,100].

Future intervention studies should also evaluate the effectiveness of explicit handwriting and graphomotor instruction. Particular attention should be given to letter formation practice, visual–motor integration, handwriting fluency training, and structured classroom-based activities designed for children at risk of handwriting difficulties [24,25,26,32,33,34]. Outcomes should include not only legibility and speed but also spelling, written composition, academic participation, perceived effort, motivation, and the need for compensatory supports.

The role of typing and assistive technologies should be investigated as part of individualized educational planning rather than as a universal replacement for handwriting. For children with persistent dysgraphia or significant neurodevelopmental difficulties, typing, word processing, text-to-speech, speech-to-text, and other digital supports may reduce transcriptional burden and promote participation [76,77,88,89]. Ongoing studies should clarify when these tools function as accommodations, when they support learning, and when they may unintentionally reduce practice in still-developing graphomotor and orthographic skills.

Finally, the relationship between handwriting, reflective writing, emotional regulation, and school mental health requires more direct empirical investigation. Current evidence supports writing-based activities more broadly, but most studies do not isolate handwriting from other writing modalities [55,56,57,58,59,60,61]. Future trials should directly compare handwritten and digital reflective writing, using developmental, cognitive, emotional, and educational outcomes. Claims regarding handwriting and mental health should therefore remain cautious until stronger modality-specific evidence is available.

## 13. Conclusions

Handwriting remains developmentally relevant in the digital school, particularly during the years in which children acquire graphomotor control, letter knowledge, spelling, orthographic representations, and written language fluency. The evidence reviewed in this article indicates that handwriting is not merely a traditional mode of text production but a complex sensorimotor and cognitive activity involving fine motor control, visual–motor integration, proprioceptive feedback, motor planning, attention, and symbolic learning [2,23,24,25,26,27,28,29,30].

The strongest evidence concerns the role of handwriting in early literacy acquisition and written language development. Handwriting practice appears particularly important when children are learning to connect visual letter forms with motor programs, phonological information, spelling, and orthographic representations [3,28,29,30]. In later stages, handwriting fluency remains relevant because slow or poorly automatized transcription may increase cognitive load and interfere with spelling, sentence generation, written composition, and text quality [32,33,34,35,41,42].

Typing and digital tools should not be considered intrinsically opposed to handwriting. Rather, they represent different modalities of written expression, each with specific developmental and educational functions. Typing may support speed, revision, accessibility, and academic productivity, especially when literacy and transcription skills are more consolidated [36,37,38,39,40,85,86]. For children with dysgraphia, specific learning disorders, ADHD, ASD, or other neurodevelopmental vulnerabilities, digital and assistive technologies may also reduce transcriptional barriers and support participation [8,9,10,76,77,78,88,89].

At the same time, compensatory technologies should not lead to the premature abandonment of handwriting instruction during the period in which graphomotor and orthographic skills are still developing. Children with handwriting difficulties require careful assessment, targeted support, and individualized educational planning. The goal should be functional access without rigidly preserving handwriting when it becomes a barrier but also without replacing it before its developmental role has been adequately supported [3,8,9,42,76,77].

The relationship between handwriting and emotional regulation or school mental health is promising but less directly established than its relationship with literacy and written language development. Writing-based activities may support reflection, emotional awareness, and meaning-making, but current evidence generally concerns writing practices rather than handwriting specifically [55,56,57,58,59,60,61]. Therefore, handwriting-based reflective activities should be considered potentially useful educational practices, not stand-alone mental health interventions.

The available evidence supports a balanced, developmentally sensitive hybrid model. Handwriting should be preserved and explicitly taught during key stages of graphomotor and literacy development (for example, preschool and primary school), while typing, tablet-based tools, and assistive technologies should be introduced according to age, learning goals, functional needs, and individual vulnerability. The central educational question is not whether handwriting or digital tools are superior, but how each modality can be used at the right developmental moment to promote and support literacy, learning, inclusion, participation, and mental health in the digital age.

## Figures and Tables

**Table 1:** Developmental, cognitive, and educational differences between handwriting, typing, and digital writing tools in school-aged children.

Domain	Handwriting	Typing	Digital/Assistive Tools	Developmental Implication
Sensorimotor engagement	High: fine motor control, visuomotor integration, proprioception	Lower, more standardized motor act	Variable; may include touch, stylus, speech-to-text	Handwriting most relevant in early literacy
Letter learning	Active construction of letter forms	Selection of preformed letters	Depends on tool	Supports orthographic encoding
Cognitive load	Initially high, then decreases with fluency	Lower transcription burden	May reduce barriers	Typing useful when handwriting is impaired
Memory/conceptual learning	More selective/elaborative processing	Faster, more verbatim	Useful for revision and access	Modality should match learning goal
Emotional/self-reflective use	Slower, embodied, materially traceable	Faster, editable	May improve privacy/accessibility	Handwritten reflection may support pacing
Vulnerable populations	Needs support in dysgraphia/ADHD	Compensatory for SLD/dysgraphia	Essential for inclusion	Avoid both premature substitution and rigid preservation

Abbreviations: ADHD, attention-deficit/hyperactivity disorder; SLD, specific learning disorder.

**Table 2:** Developmentally informed recommendations for integrating handwriting and digital tools in school mental health promotion.

Population/Stage	Main Developmental Need	Potential Risk of Excessive Digital Substitution	Role of Graphomotor Exercises	Role of Digital Tools	Practical Recommendation
Preschool children	Sensorimotor exploration, pre-writing skills, emergent literacy	Displacement of play, manipulation, drawing, movement, and face-to-face interaction	Drawing, tracing, copying simple shapes, pre-writing activities, and early letter formation to support fine motor control and visual–motor integration [24,25,26]	Limited, adult-mediated, interactive, and developmentally appropriate use	Preserve non-screen sensorimotor activities and introduce digital tools only as guided supports [11,14,19]
Early primary school	Letter learning, spelling, orthographic learning, and handwriting automatization	Premature reduction in graphomotor practice during literacy acquisition	Explicit letter formation practice, handwriting practice, and handwriting fluency exercises to support letter recognition and orthographic learning [3,28,29,30,32,33,34,35]	Gradual introduction of digital tools for specific learning goals	Do not replace handwriting instruction before graphomotor and orthographic skills are sufficiently consolidated [3,28,29,30]
Late primary school	Written fluency, written composition, note-taking, and revision	Overreliance on copy/paste, passive digital use, or reduced handwriting fluency	Handwriting fluency exercises, written summaries, and structured handwritten notes when the goal is conceptual encoding and written organization [32,33,34,35,36,37,38,41,42]	Typing and word processing for drafting, revision, projects, and accessibility	Match writing modality to task demands, balancing handwriting for learning and typing for revision or productivity [36,37,38,39,40,85,86,87]
Adolescents	Conceptual learning, self-reflection, academic autonomy, and self-regulated writing	Multitasking, sleep disruption, social comparison, and fragmented attention	Longhand note-taking, reflective writing, and structured writing activities when the goal is elaboration, synthesis, or reflective pacing [36,37,38,39,53,54,55,56,57,58,59,60,61]	Collaborative tools, assistive tools, and digital platforms used with clear goals and limits	Promote regulated hybrid use and avoid dysregulated digital engagement [1,13,62,63,64,70,71,72]
ADHD/attentional vulnerability	Attention, inhibition, task persistence, and self-regulation	Overstimulation, reward-driven use, multitasking, and sleep disruption	Short, structured, and predictable graphomotor routines with reduced distraction and clear feedback [7,21,22,78]	Distraction-limited digital tools, visual supports, and assistive functions	Combine handwriting support with digital regulation and environmental scaffolding [7,21,22,78]
Autism spectrum disorder	Structured learning, communication, flexibility, and participation	Reinforcement of solitary, rigid, or poorly mediated routines	Drawing or writing tasks when tolerated, individualized according to sensory profile, motor abilities, and communication goals [10,82]	Visual supports, augmentative and alternative communication, structured apps, and assistive technologies	Use technology as a structured support, not as an automatic substitute for embodied learning [10,82]
Specific learning disorders/dysgraphia	Access to written expression, handwriting quality, fluency, and participation	Avoiding graphomotor development entirely or delaying needed accommodations	Targeted handwriting remediation, graphomotor training, and handwriting fluency support when appropriate [8,9,76,77]	Typing, word processing, text-to-speech, speech-to-text, and other assistive technologies	Combine functional access with individualized remediation; avoid both premature abandonment and rigid preservation of handwriting [8,9,76,77,88,89]

## Data Availability

No new data were created or analyzed in this study. Data sharing is not applicable to this article.
